# Preoperative Inflammatory Markers in Relation to the Malignant Potential of Cartilaginous Tumors: A Retrospective Cohort Study

**DOI:** 10.3390/jcm15207885

**Published:** 2026-10-13

**Authors:** İbrahim Halil Demir, Mustafa Onur Karaca, Rıdvan Acar, Mustafa Özyıldıran, Hüseyin Yusuf Yıldız, İdris Perktaş, Bilgin Bozgeyik, Nevzat Gönder

**Affiliations:** 1 Department of Orthopaedics and Traumatology, Gaziantep City Hospital, Gaziantep 27500, Türkiye; 2 Department of Orthopaedics and Traumatology, Ankara University School of Medicine, Ankara 06230, Türkiye; 3 Department of Orthopaedics and Traumatology, Sanliurfa Training and Research Hospital, Sanliurfa 63250, Türkiye; 4 Department of Orthopaedics and Traumatology, Sandikli State Hospital, Afyonkarahisar 03500, Türkiye; 5 Department of Orthopaedics and Traumatology, Gaziantep University School of Medicine, Gaziantep 27310, Türkiye

**Keywords:** cartilage tumors, inflammatory markers, inflammatory indices, tumor grading, pan-immune-inflammation value

## Abstract

**Background:** The definitive diagnosis of cartilaginous tumors remains challenging because imaging and biopsy findings may not reliably distinguish low-grade from high-grade lesions. Consequently, the final diagnosis is often established only after surgical excision and histopathological examination. Inflammatory biomarkers derived from routine peripheral blood tests have emerged as potential adjunctive tools for tumor characterization, but their role in cartilaginous tumors remains unclear. **Questions/Purposes:** (1) Are preoperative inflammatory markers different between low-grade and high-grade cartilaginous tumors? (2) Which inflammatory markers are associated with high-grade (versus low-grade) cartilaginous tumors? (3) What is the diagnostic performance of the pan-immune inflammation value (PIV) in predicting high-grade cartilaginous tumors? **Patients and Methods:** We retrospectively reviewed 67 patients who underwent surgery for cartilaginous tumors between January 2022 and July 2025 and received definitive histopathological diagnoses. Patients were divided into two groups according to tumor grade. Group A consisted of enchondroma (n = 9), atypical cartilaginous tumor (ACT) (n = 13), and Grade 1 chondrosarcoma (n = 14), whereas Group B consisted of Grade 2 (n = 19) and Grade 3 chondrosarcoma (n = 12). Preoperative peripheral blood inflammatory markers, including white blood cell count (WBC), erythrocyte sedimentation rate (ESR), C-reactive protein (CRP), mean platelet volume (MPV), red blood cell distribution width (RDW), neutrophil-to-lymphocyte ratio (NLR), platelet-to-lymphocyte ratio (PLR), systemic immune-inflammation index (SII), systemic inflammation response index (SIRI), and pan-immune inflammation value (PIV), were compared between groups. Receiver operating characteristic (ROC) analysis was performed to evaluate the diagnostic performance of significant biomarkers. **Results:** Baseline demographic and clinical characteristics were comparable between groups. Patients with high-grade chondrosarcoma (Group B) demonstrated higher CRP (6.35 ± 5.25 versus 4.02 ± 3.33; *p* = 0.030), PLR (198.08 ± 98.83 versus 139.11 ± 73.76; *p* = 0.016), SII (728.33 ± 177.26 versus 526.50 ± 126.17; *p* = 0.001), SIRI (2.01 ± 0.72 versus 1.40 ± 0.62; *p* = 0.001), and PIV (629.68 ± 125.19 versus 295.00 ± 111.48; *p* = 0.001) compared with Group A. ROC analysis showed that PIV had the highest diagnostic accuracy, with a cutoff value greater than 461.5 yielding a sensitivity of 96.8% and a specificity of 97.2% for identifying high-grade malignancy (*p* < 0.001). **Conclusions:** Preoperative inflammatory markers, particularly SII, SIRI, and PIV, were associated with high-grade cartilaginous tumors; differences in CRP and PLR did not survive correction for multiple comparisons. Among these markers, PIV demonstrated excellent discriminatory ability for distinguishing high-grade from low-grade cartilaginous tumors. These findings suggest that PIV may serve as a readily available and low-cost adjunctive biomarker in the preoperative evaluation of cartilaginous tumors. Further prospective studies are warranted to validate its clinical utility and determine its role alongside established radiological and histopathological assessment methods. **Level of Evidence:** Level III, diagnostic study (retrospective comparative cohort study).

## 1. Introduction

Cartilaginous tumors represent a heterogeneous group of bone neoplasms ranging from benign enchondromas to high-grade chondrosarcomas [1]. Accurate preoperative differentiation between low-grade and high-grade lesions remains a major clinical challenge due to overlapping radiological and histopathological features [2,3]. In particular, distinguishing atypical cartilaginous tumors and low-grade chondrosarcomas from high-grade malignant tumors is often difficult, yet crucial for determining optimal surgical management and predicting prognosis [4].

The diagnostic evaluation of cartilage tumors is primarily based on clinical assessment, imaging methods, and histopathological examination [5]. While advanced imaging techniques such as magnetic resonance imaging and computed tomography provide valuable information about tumor characteristics, they may not reliably predict the grade or biological behaviour of the tumor [5,6]. Furthermore, since cartilage tumors are heterogeneous, biopsy may not yield clear and reliable diagnostic results [7]. Consequently, a definitive diagnosis is usually only made after surgical excision and detailed pathological analysis, which can delay appropriate treatment planning and expose patients to unnecessary procedures.

Recent studies have provided increasing evidence regarding the role of systemic inflammation in tumor development, progression, and metastasis [8,9,10]. Inflammatory cells and mediators in the tumor microenvironment contribute to angiogenesis, immune evasion, and cellular proliferation [11]. Accordingly, peripheral blood-based inflammatory markers have emerged as accessible, cost-effective, and reproducible biomarkers for cancer diagnosis and prognosis. Parameters such as C-reactive protein (CRP), neutrophil-to-lymphocyte ratio (NLR), pan-immune-inflammation value (PIV), systemic immune-inflammation index (SII), and systemic inflammation response index (SIRI) have been investigated in many cancer types and have shown predictive value in malignancy and prognosis [10,12,13].

Studies investigating the relationship between inflammatory markers and tumor aggressiveness, particularly in cartilage tumors, are limited. Identifying preoperative biomarkers that can differentiate between low-grade and high-grade cartilage tumors could significantly improve diagnostic accuracy and guide clinicians in developing individualized treatment strategies.

The aim of the present study is to evaluate the diagnostic value of preoperative peripheral blood inflammatory markers in patients with surgically treated cartilage tumors. By comparing inflammatory parameters between low-grade and high-grade lesions, we aimed to determine their potential roles in predicting malignancy and to identify practical biomarkers that can support clinical decision-making in the preoperative period.

## 2. Materials and Methods

### 2.1. Study Design and Population

This retrospective study was conducted after obtaining approval from the local Institutional Review Board. As this was a retrospective review of existing clinical records, the requirement for individual informed consent was waived by the Institutional Review Board, and the study was conducted in accordance with the principles of the Declaration of Helsinki. Patients who underwent surgical treatment for cartilaginous tumors at our institution between January 2022 and July 2025 were retrospectively reviewed. Preoperative routine laboratory tests, including inflammatory markers, were compared with postoperative histopathological findings. Patients with histopathological diagnoses of cartilaginous tumors, including enchondroma, atypical cartilaginous tumor (ACT), and chondrosarcoma grades 1, 2, and 3, were included in the study.

**Figure 1:**
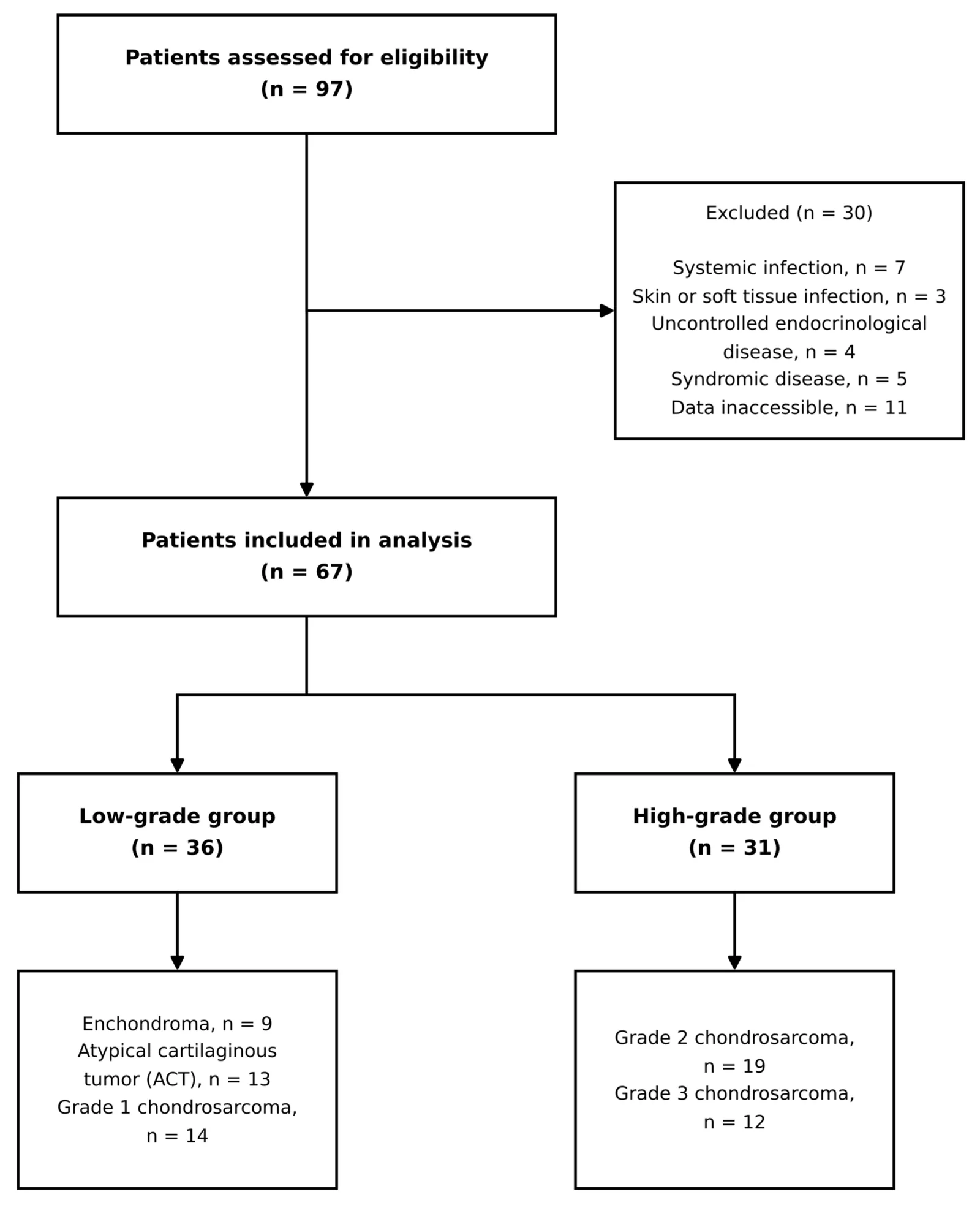
Patient flow diagram showing the number of patients screened, excluded (by reason), and included in the low-grade and high-grade analysis groups, with the underlying histopathological diagnoses in each group.

Patients with evidence of systemic or local (skin or soft tissue) infection within two weeks prior to preoperative blood sampling, uncontrolled endocrinological disease, syndromic disease, or incomplete/inaccessible clinical data were excluded to minimize potential confounding effects on inflammatory parameters and to ensure data completeness. Of 97 patients initially screened, 30 were excluded (systemic infection, n = 7; skin or soft tissue infection, n = 3; uncontrolled endocrinological disease, n = 4; syndromic disease, n = 5; data inaccessible, n = 11), and a total of 67 patients met the inclusion criteria and were enrolled in the study (Figure 1).

### 2.2. Group Classification

Based on histopathological findings, patients were stratified into two groups. Group A (low-grade group) consisted of patients diagnosed with enchondroma (n = 9), ACT (n = 13), and grade 1 chondrosarcoma (n = 14). Group B (high-grade group) included patients with grade 2 (n = 19) and grade 3 (n = 12) chondrosarcoma.

This classification was adopted because grade 2 and grade 3 chondrosarcomas are considered high-grade tumors that typically require wide surgical resection. Patients with other primary bone tumors, metastatic bone disease or cartilage tumors presenting with distant metastases at diagnosis were excluded from the study. Histopathological grading of chondrosarcoma (Grades 1–3) was assigned by the institution’s pathologists based on the surgical pathology reports, using the World Health Organization Classification of Soft Tissue and Bone Tumours (5th edition, 2020).

### 2.3. Data Collection

Demographic and clinical data including sex, age, body mass index (BMI), smoking status, presence of diabetes, tumor location (upper or lower extremity), and pathological diagnosis were recorded. Pre-biopsy blood samples obtained during preoperative evaluation were analyzed for inflammatory and hematological parameters. All samples were drawn before any diagnostic biopsy or surgical intervention, so as to avoid potential confounding of inflammatory parameters by a preceding invasive procedure. The following laboratory values were recorded:

- WBC (White Blood Cell Count; 10^9^/L, reference: 4.5–11).
- ESR (Erythrocyte Sedimentation Rate; mm/h, reference: 0–15).
- CRP (C-reactive Protein; mg/L, reference: 0–5).
- MPV (Mean Platelet Volume; fL, reference: 9.2–12.1).
- RDW (Red Cell Distribution Width; %, reference: 11.5–14.5).
- NLR (Neutrophil-to-Lymphocyte Ratio; neutrophil count [10^9^/L] ÷ lymphocyte count [10^9^/L]).
- PLR (Platelet-to-Lymphocyte Ratio; platelet count [10^9^/L] ÷ lymphocyte count [10^9^/L]).
- SII (Systemic Immune-Inflammation Index; neutrophil count [10^9^/L] × platelet count [10^9^/L] ÷ lymphocyte count [10^9^/L]).
- SIRI (Systemic Inflammation Response Index; neutrophil count [10^9^/L] × monocyte count [10^9^/L] ÷ lymphocyte count [10^9^/L]).
- PIV (Pan-Immune-Inflammation Value; neutrophil count [10^9^/L] × platelet count [10^9^/L] × monocyte count [10^9^/L] ÷ lymphocyte count [10^9^/L]).

These parameters were compared between patients with low-grade and high-grade cartilage tumors according to their final pathological diagnoses.

### 2.4. Statistical Analysis

The normality of continuous variables was assessed using the Shapiro–Wilk test. Normally distributed variables were compared between two groups using Student’s *t*-test, whereas non-normally distributed variables were analyzed using the Mann–Whitney U test. Associations between categorical variables were evaluated using the chi-square test. Receiver operating characteristic (ROC) curve analysis was performed to determine optimal cutoff values for the studied parameters. Optimal cut-off values were determined using the Youden index. Statistical analyses were conducted using SPSS software (version 22.0 for Windows; IBM Corp., Armonk, NY, USA) and MedCalc statistical software (version 23.3.7; MedCalc Software Ltd., Ostend, Belgium). A *p*-value < 0.05 was considered statistically significant. Because ten inflammatory markers were compared between groups, a Bonferroni-corrected significance threshold (α = 0.005) was additionally applied post hoc to assess whether the observed differences survived correction for multiplicity. Positive and negative predictive values for PIV were calculated from its sensitivity, specificity, and the observed prevalence of high-grade tumors in the cohort. To assess whether PIV provided diagnostic information beyond routinely available clinical variables, multivariable logistic regression models incorporating age, sex, smoking status, comorbidity, BMI, and tumor location, with and without PIV, were compared using the likelihood ratio test; because PIV induced quasi-complete separation between groups, Firth’s penalized logistic regression was used for this analysis. Internal validation of the ROC-derived cutoff was performed using a bootstrap optimism-correction procedure (2000 resamples): for each bootstrap sample, the Youden-optimal cutoff was re-derived and its performance evaluated on the original cohort, yielding optimism-corrected estimates of sensitivity, specificity, and AUC. Differences in PIV, SII, and SIRI across the five underlying histopathological diagnoses (enchondroma, ACT, and chondrosarcoma grades 1–3) were additionally explored using the Kruskal–Wallis test with Dunn’s post hoc test and Bonferroni adjustment.

## 3. Results

A total of 67 patients who met the inclusion criteria were enrolled in the study and divided into low-grade and high-grade groups. In this retrospectively designed study, patients diagnosed postoperatively with enchondroma (n = 9), ACT (n = 13), and grade 1 chondrosarcoma (n = 14) were classified into the low-grade group (n = 36), whereas patients with pathological diagnoses of grade 2 chondrosarcoma (n = 19) and grade 3 chondrosarcoma (n = 12) were classified into the high-grade group (n = 31). No statistically significant differences were observed between the low-grade and high-grade groups in terms of demographic variables, including sex, age, BMI, smoking status, comorbidities, and tumor location (upper or lower extremity) (Table 1).

Preoperative hematological and laboratory parameters, including WBC, ESR, CRP, MPV, RDW, NLR, PLR, SII, SIRI, and PIV, were compared between the two groups. While no statistically significant differences were observed in WBC, ESR, MPV, RDW, and NLR values, CRP (4.02 ± 3.33 vs. 6.35 ± 5.25; *p* = 0.030), PLR (139.11 ± 73.76 vs. 198.08 ± 98.83; *p* = 0.016), SII (526.50 ± 126.17 vs. 728.33 ± 177.26; *p* = 0.001), SIRI (1.40 ± 0.62 vs. 2.01 ± 0.72; *p* = 0.001), and PIV (295.00 ± 111.48 vs. 629.68 ± 125.19; *p* = 0.001) were significantly higher in the high-grade group (Table 2). After applying a Bonferroni correction for multiple comparisons (corrected α = 0.005), the differences in SII, SIRI, and PIV remained statistically significant, whereas the differences in CRP and PLR did not survive correction for multiplicity.

**Figure 2:**
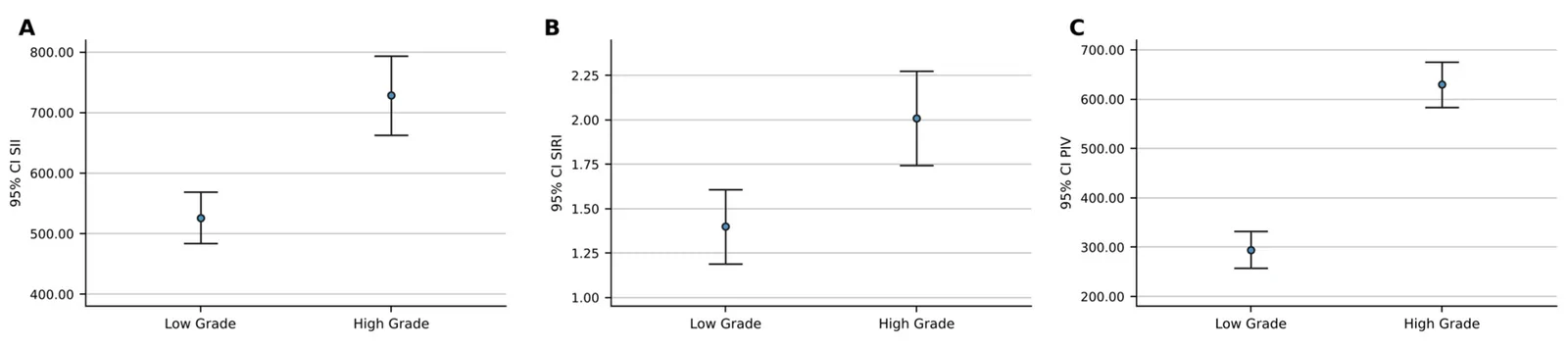
Comparison of (**A**) systemic immune-inflammation index (SII), (**B**) systemic inflammation response index (SIRI), and (**C**) pan-immune-inflammation value (PIV) between low- and high-grade cartilage tumors, shown as error bars with 95% confidence intervals.

Notably, SII, SIRI, and PIV recently highlighted in the literature as prominent inflammation-based biomarkers were also significantly higher in the high-grade group in our cohort (Figure 2). Receiver operating characteristic (ROC) curve analysis demonstrated that PIV had a cutoff value of >461.5, with a sensitivity of 96.77% and a specificity of 97.22% (*p* < 0.001). SII showed a cutoff value of >599, with a sensitivity of 87.10% and a specificity of 72.22% (*p* < 0.001), while SIRI demonstrated a cutoff value of >1.16, with a sensitivity of 93.55% and a specificity of 50.00% (*p* = 0.002) for predicting high-grade malignancy in cartilaginous tumors (Table 3). Based on the observed sensitivity, specificity, and disease prevalence in this cohort (46.3% high-grade), the positive and negative predictive values were 96.8% and 97.2% for PIV, 73.0% and 86.7% for SII, and 61.7% and 90.0% for SIRI, respectively (Table 3).

**Figure 3:**
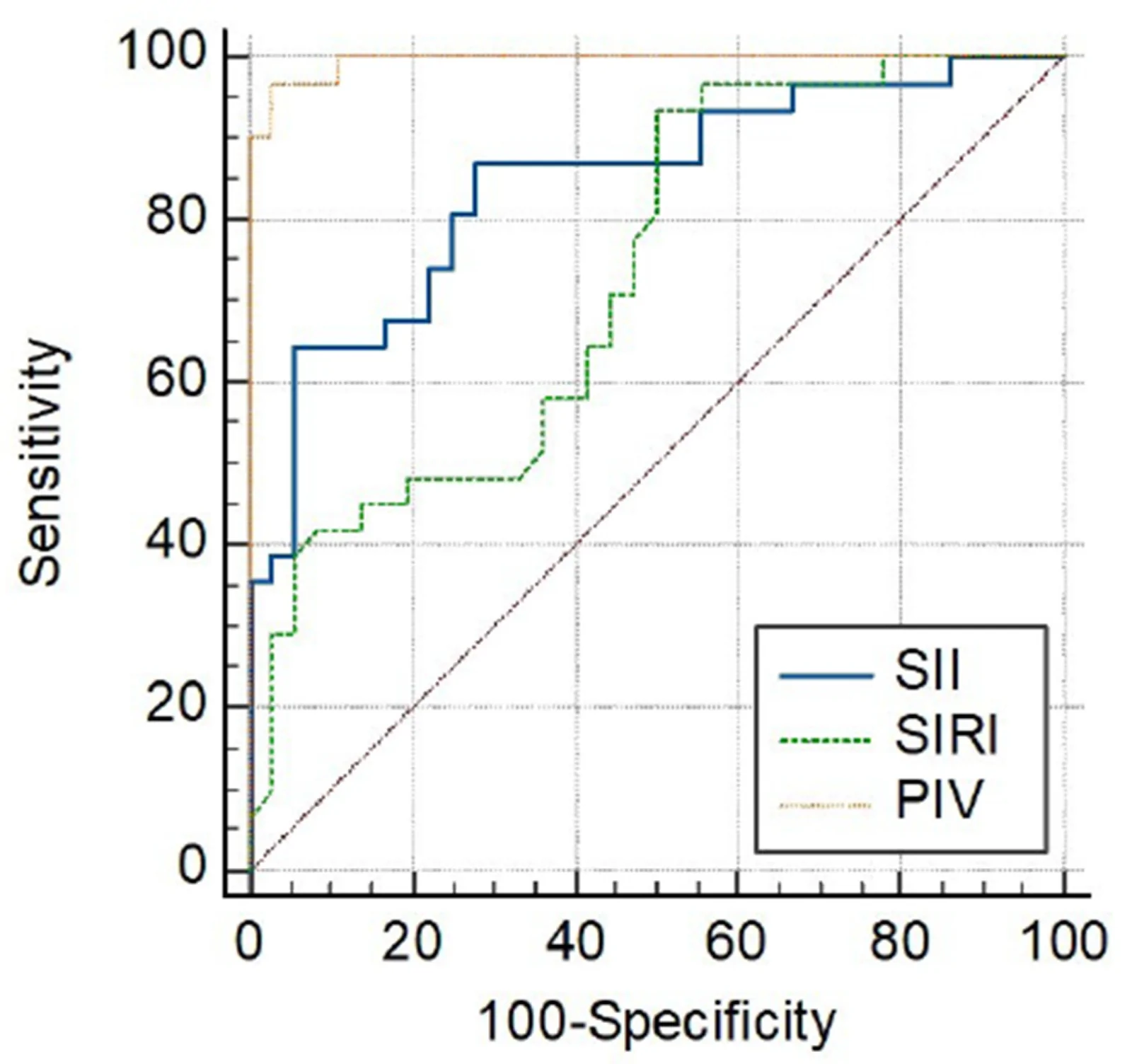
Receiver operating characteristic (ROC) curves plotted for the prediction of high-grade (versus low-grade) cartilaginous tumors using PIV, SII, and SIRI. The diagonal dotted line represents the reference line (AUC = 0.5). Abbreviations: AUC, area under the curve; PIV, pan-immune-inflammation value; SII, systemic immune-inflammation index; SIRI, systemic inflammation response index.

Comparison of the areas under the curve (AUCs) obtained from ROC analyses for PIV, SII, and SIRI in predicting high-grade malignancy revealed that PIV was significantly superior to both SII (*p* = 0.0019) and SIRI (*p* < 0.0001). No statistically significant difference was observed between SII and SIRI (*p* = 0.0989) (Table 4; Figure 3).

In multivariable Firth logistic regression, PIV remained strongly associated with high-grade malignancy after adjustment for age, sex, smoking status, comorbidity, BMI, and tumor location (likelihood ratio test comparing models with and without PIV: χ^2^ = 64.5, df = 1, *p* < 0.001); none of the clinical covariates were independently associated with tumor grade. Because PIV separated the two groups almost completely, the odds ratio estimate from this model was numerically unstable (point estimate with a very wide confidence interval) despite the highly significant likelihood ratio test, itself illustrating the separation/overfitting concern discussed below (Table 5). Bootstrap internal validation (2000 resamples) of the Youden-optimal cutoff yielded an optimism-corrected sensitivity of 90.9% and specificity of 96.1% for PIV (apparent values 96.8% and 97.2%, respectively), with negligible optimism in the AUC itself (optimism-corrected AUC 0.995). Across the five underlying histopathological diagnoses, PIV increased progressively from enchondroma (159.1 ± 40.1) to ACT (296.6 ± 87.6), grade 1 (380.9 ± 68.1), grade 2 (546.6 ± 65.6), and grade 3 chondrosarcoma (761.2 ± 70.2) (Kruskal–Wallis *p* < 0.001); after Bonferroni-adjusted pairwise (Dunn) comparisons, PIV did not differ significantly among enchondroma, ACT, and grade 1 chondrosarcoma (all *p* > 0.05), and did not differ significantly between grade 2 and grade 3 chondrosarcoma (*p* = 0.305). Every comparison between a low-grade entity (enchondroma, ACT, or grade 1) and a high-grade entity (grade 2 or grade 3) reached statistical significance (all *p* ≤ 0.001), with the sole exception of the grade 1 versus grade 2 comparison, which approached but did not reach significance (*p* = 0.059) (Table 6). This pattern is consistent with, and lends empirical support to, our a priori grouping of enchondroma, ACT, and grade 1 chondrosarcoma as the low-grade category.

## 4. Discussion

The accurate diagnosis and management of cartilaginous tumors is a complex process that requires a specialized multidisciplinary team comprising musculoskeletal pathologists, radiologists, and orthopedic oncologists [14,15]. However, outside of major referral centers, the establishment of such specialized teams is uncommon in most hospitals [16]. Due to the heterogeneous nature of cartilaginous tumors, biopsy-based diagnosis is often challenging, and radiological assessment followed by surgical excision of the lesion is frequently required for definitive diagnosis [9,17]. Inaccurate diagnosis and inappropriate excision of cartilaginous tumors may result in limb loss and may even pose a life-threatening risk to the patient [2,18]. In such critical situations, clinicians require additional preoperative indicators to support the confirmation of malignancy. Therefore, the present study aimed to evaluate the potential utility of readily available peripheral blood inflammatory markers as supportive tools for distinguishing malignant from benign cartilaginous tumors.

Our findings are consistent with the existing literature emphasizing the prognostic and diagnostic significance of systemic inflammation in oncology. The inflammatory markers evaluated in our study have previously been associated with tumor aggressiveness in soft tissue sarcomas [19], survival outcomes in osteosarcoma [20], differential diagnosis in uterine tumors [21], and prognosis in breast cancer [22]. Recently developed composite indices, including SII, SIRI, and PIV, have demonstrated promising value in predicting outcomes in various carcinomas [12,21,22,23]. To the best of our knowledge, this study represents the first comprehensive evaluation of an extended panel of inflammatory indices, including SII, SIRI, and PIV, specifically for predicting malignancy in cartilaginous tumors.

C-reactive protein, one of the most important indicators of the immune response, and the platelet-to-lymphocyte ratio, which reflects platelet activation and inflammatory activity, may indicate a pro-thrombotic and pro-inflammatory state associated with malignancy in cartilaginous tumors and may therefore contribute to malignancy prediction and prognostic assessment [24,25]. More importantly, composite indices integrating neutrophil, platelet, monocyte, and lymphocyte counts—such as SII, SIRI, and PIV—may provide a more comprehensive evaluation of the immune-inflammatory microenvironment than conventional inflammatory individual parameters. In particular, PIV appears to function as a strong predictor of malignancy [21,26,27]. Our study demonstrated that SII (>599), SIRI (>1.16), and PIV (>461.5) may be used as predictive markers for high-grade malignancy in cartilaginous tumors. Notably, elevated PIVs were associated with high sensitivity (96.77%) and specificity (97.22%), highlighting their potential clinical utility.

It is important to note that SII, SIRI, and PIV are not fully independent biomarkers, as they are mathematically derived from overlapping cellular components—neutrophil, platelet, monocyte, and lymphocyte counts. In particular, PIV is calculated as SIRI multiplied by the platelet count. Consequently, the superior performance of PIV over SIRI observed in our ROC comparison (Table 4) may partly reflect a difference in platelet counts between groups rather than an entirely independent diagnostic contribution. This mathematical interrelationship should be considered when interpreting the comparative performance of these composite indices, and future studies should report the individual cell counts alongside the composite indices to clarify the incremental value of each.

We also acknowledge that our two-group classification, which combined enchondroma, atypical cartilaginous tumor, and grade 1 chondrosarcoma into a single low-grade category, groups together biologically and clinically distinct entities. This classification reflects the shared surgical decision threshold of these lesions relative to grade 2/3 chondrosarcoma, which typically requires wide resection, but it does not, by itself, capture differences in metastatic potential and treatment intensity within the low-grade group. To explore this further, we compared PIV, SII, and SIRI across the five underlying diagnoses directly. PIV increased progressively from enchondroma through ACT, grade 1, grade 2, and grade 3 chondrosarcoma, and post hoc pairwise comparisons showed no significant differences among enchondroma, ACT, and grade 1 chondrosarcoma, nor between grade 2 and grade 3 chondrosarcoma, while every comparison between the low-grade and high-grade entities was significant except grade 1 versus grade 2, which approached but did not reach significance (*p* = 0.059) (Table 6). This pattern provides empirical, data-driven support for our a priori low-grade versus high-grade dichotomization, although the modest number of patients in each individual diagnostic subgroup means these pairwise comparisons should be regarded as exploratory, and larger cohorts are needed to confirm whether inflammatory markers can meaningfully separate enchondroma from ACT/grade 1 chondrosarcoma.

A critical consideration is the confounding effect of concurrent infection or systemic inflammation, which was minimized in our study by excluding patients with recent infections. Therefore, these biomarkers should always be interpreted within the broader clinical and radiological context.

### Limitations

This study has several important limitations. First, its retrospective, single-center design and modest sample size (n = 67, with only 31 patients in the high-grade group) predispose the ROC analysis to overfitting; no a priori sample size or power calculation was performed, and the very high apparent AUC (0.995) for PIV should be interpreted with caution given the limited number of events. Second, because the reported cutoff was derived and tested on the same dataset, we performed a bootstrap optimism-correction procedure (2000 resamples); this reduced the sensitivity of PIV from an apparent 96.8% to an optimism-corrected 90.9% (specificity 97.2% to 96.1%), confirming that the originally reported performance was somewhat optimistic, although the corrected performance remained high. External validation in an independent cohort is still required before clinical application. Third, ten inflammatory markers were compared without correction for multiplicity in the primary analysis; a post hoc Bonferroni correction confirmed that SII, SIRI, and PIV remained significant, whereas CRP and PLR did not, and the latter findings should be regarded as hypothesis-generating rather than confirmatory. Fourth, as discussed above, SII, SIRI, and PIV are mathematically interrelated composite indices derived from overlapping cell counts, which limits their interpretation as fully independent biomarkers. Fifth, our two-group classification combined enchondroma, ACT, and grade 1 chondrosarcoma into a single low-grade category; subgroup analysis across the five underlying diagnoses provided empirical support for this grouping (no significant pairwise differences in PIV among enchondroma, ACT, and grade 1 chondrosarcoma after correction for multiple comparisons), but individual subgroup sizes were small and this analysis should be considered exploratory. Sixth, on multivariable Firth logistic regression, PIV remained strongly associated with tumor grade after adjustment for age, sex, smoking status, comorbidity, BMI, and tumor location (likelihood ratio test *p* < 0.001); however, because PIV nearly completely separated the two groups, the corresponding odds ratio estimate was numerically unstable, and this analysis did not include radiological variables, which were not available in our dataset. Seventh, we were unable to provide a reliable comparison of anatomical site or maximum tumor diameter across the diagnostic groups: because the low-grade group was predominantly managed with intralesional curettage and the high-grade group with wide surgical resection, the extent of tissue available for measurement differed systematically between groups, precluding a consistent size assessment. Because PIV may in principle reflect tumor burden rather than histological grade per se, this remains an important question that should be addressed in future prospective studies using standardized preoperative volumetric imaging. Patients with uncontrolled endocrinological disease (n = 4) and syndromic disease (n = 5) were excluded (see Figure 1); however, we did not systematically exclude patients with active/treated chronic inflammatory or autoimmune conditions, nor did we record discordance rates between radiological, biopsy, and final histopathological diagnoses; both factors should be addressed in future prospective, multicenter studies designed to externally validate these findings before their incorporation into standardized clinical pathways.

## 5. Conclusions

Preoperative inflammatory markers, particularly PIV, SII, and SIRI, represent practical and cost-effective tools for distinguishing high-grade from low-grade cartilaginous tumors; CRP and PLR differences did not survive correction for multiple comparisons and should be regarded as hypothesis-generating. Among these, PIV demonstrates the strongest discriminatory performance, with high sensitivity and specificity in this cohort. Pending prospective, external validation to address the overfitting risk discussed above, incorporation of these indices into routine preoperative assessment may improve diagnostic accuracy, optimize surgical planning, and strengthen multidisciplinary decision-making in clinically ambiguous cases. Further prospective, multicenter studies are required to validate these findings and support their integration into standardized clinical pathways.

## Figures and Tables

**Table 1:** Demographics and Patient Characteristics.

		Cartilage Tumors		*p* Value
		Low Grade	High Grade	
		(n = 36)	(n = 31)	
Age (year)		45.69 ± 15.25	50.74 ± 11.93	0.141
BMI (kg/m^2^)		26.14 ± 2.18	25.41 ± 2.83	0.238
Sex	Female	17 (47.2%)	15 (48.4%)	0.924
	Male	19 (52.8%)	16 (51.6%)	
Smoking	No	26 (72.2%)	18 (58.1%)	0.224
	Yes	10 (27.8%)	13 (41.9%)	
Comorbidity	No	27 (75.0%)	21 (67.7%)	0.511
	Yes	9 (25.0%)	10 (32.3%)	
Extremity	Upper	13 (36.1%)	10 (32.3%)	0.74
	Lower	23 (63.9%)	21 (67.7%)	

The Mann–Whitney U test was used to compare numerical data. The chi-square test or Fisher’s exact test was used for categorical data. Values are presented as mean ± standard deviation or number (%). A *p*-value < 0.05 was considered statistically significant. Abbreviations: BMI, body mass index.

**Table 2:** Comparison of Inflammatory Markers in Patients with Cartilage Tumors.

Cartilage Tumors					
	Low Grade		High Grade		*p* Value
	Mean ± SD	Median (%25–%75)	Mean ± SD	Median (%25–%75)	
WBC	8.13 ± 1.82	8.02 (6.59–8.98)	7.92 ± 2.14	7.7 (6.2–9.4)	0.668
ESR	8.28 ± 5.53	6.5 (4–12)	8.13 ± 5.09	7 (4–12)	0.945
CRP	4.02 ± 3.33	3.05 (1.8–4.55)	6.35 ± 5.25	4.1 (2.7–9.7)	0.030 *
MPV	9.33 ± 1.54	9.5 (9–9.7)	9.61 ± 0.82	9.6 (8.8–10.3)	0.614
NLR	2.23 ± 0.9	1.96 (1.61–2.56)	2.42 ± 0.87	2.18 (1.84–2.72)	0.172
RDW	13.41 ± 0.83	13.5 (12.9–13.95)	13.16 ± 0.88	13.3 (12.2–13.6)	0.242
PLR	139.11 ± 73.76	114.75 (96.9–149.3)	198.08 ± 98.83	182.9 (108.8–286)	0.016 *
SII	526.5 ± 126.17	541.35 (431.1–625.85)	728.33 ± 177.26	718.4 (620.4–821.4)	0.001 *
SIRI	1.4 ± 0.62	1.29 (1–1.94)	2.01 ± 0.72	1.76 (1.43–2.64)	0.001 *
PIV	295 ± 111.48	308.35 (197.9–389.45)	629.68 ± 125.19	590.6 (514.5–740.5)	0.001 *

The Mann–Whitney U test was used for the comparison of numerical data. * Statistically significant *p*-values (*p* < 0.05) are shown in bold. Abbreviations: CRP, C-reactive protein; ESR, erythrocyte sedimentation rate; MPV, mean platelet volume; NLR, neutrophil-to-lymphocyte ratio; PIV, pan-immune-inflammation value; PLR, platelet-to-lymphocyte ratio; RDW, red cell distribution width; SD, standard deviation; SII, systemic immune-inflammation index; SIRI, systemic inflammation response index; WBC, white blood cell count.

**Table 3:** Receiver operating characteristic (ROC) analysis of PIV, SII, and SIRI for differentiating high-grade from low-grade cartilaginous tumors.

	AUC (95% CI)	*p* Value	Cut-Off Value	Sensitivity (%)	Specificity (%)
PIV	0.995 (0.936–1.000)	**<0.0001**	461.5	96.77	97.22
SII	0.846 (0.737–0.922)	**<0.0001**	599	87.10	72.22
SIRI	0.732 (0.610–0.833)	**0.0002**	1.16	93.55	50.00

Receiver operating characteristic (ROC) analysis was performed to evaluate the predictive ability of pre-biopsy inflammatory indices for differentiating high-grade from low-grade cartilaginous tumors. Optimal cut-off values were determined using the Youden index. Statistically significant *p*-values (*p* < 0.05) are shown in bold. Abbreviations: AUC, area under the receiver operating characteristic curve; CI, confidence interval; PIV, pan-immune-inflammation value; SII, systemic immune-inflammation index; SIRI, systemic inflammation response index.

**Table 4:** Pairwise comparison of AUCs derived from ROC analysis of PIV, SII, and SIRI for differentiating high-grade from low-grade cartilaginous tumors.

**PIV ~ SII**	
Difference between areas	0.149
Standard Error (SE)	0.0479
95% Confidence Interval	0.0549 to 0.243
z statistic	3.106
Significance level	***p* = 0.0019**
**PIV ~ SIRI**	
Difference between areas	0.263
Standard Error (SE)	0.0609
95% Confidence Interval	0.143 to 0.382
z statistic	4.314
Significance level	***p* < 0.0001**
**SII ~ SIRI**	
Difference between areas	0.114
Standard Error (SE)	0.069
95% Confidence Interval	−0.0214 to 0.249
z statistic	1.65
Significance level	*p* = 0.0989

AUCs were compared pairwise using the DeLong method; SE indicates the standard error of the difference between AUCs. Statistically significant *p*-values (*p* < 0.05) are shown in bold. Abbreviations: AUC, area under the receiver operating characteristic curve; PIV, pan-immune-inflammation value; SE, standard error; SII, systemic immune-inflammation index; SIRI, systemic inflammation response index.

**Table 5:** Multivariable Firth’s penalized logistic regression for the prediction of high-grade cartilaginous tumors.

Variable	Coefficient (β)	SE	Odds Ratio (95% CI)	*p* Value
Age (years)	0.176	0.093	1.19 (0.99–1.43)	0.058
Sex (male)	−0.287	1.252	0.75 (0.06–9.01)	0.819
Smoking	−1.834	2.034	0.16 (0.003–8.51)	0.367
Comorbidity	−2.623	2.052	0.07 (0.001–4.09)	0.201
BMI (kg/m^2^)	−0.109	0.269	0.90 (0.53–1.52)	0.686
Extremity (lower)	−0.425	1.351	0.65 (0.05–8.99)	0.753
PIV (per 1-SD increase) *	8.993	3.066	8046.6 (19.8–3,277,338)	**0.003**

Firth’s penalized logistic regression was used because PIV induced quasi-complete separation between low- and high-grade groups in standard logistic regression. Likelihood ratio test comparing the model with PIV to the clinical-variables-only model: χ^2^ = 64.5, df = 1, *p* < 0.001. * The extremely wide confidence interval for the PIV odds ratio reflects near-complete separation between groups and should not be interpreted as a stable effect-size estimate; the highly significant likelihood ratio test is the more robust indicator that PIV contributes diagnostic information beyond the clinical variables included in the model. Statistically significant p-values (p < 0.05) are shown in bold. Abbreviations: BMI, body mass index; CI, confidence interval; PIV, pan-immune-inflammation value; SD, standard deviation; SE, standard error.

**Table 6:** PIV distribution and post hoc pairwise comparisons across the five histopathological diagnostic groups.

Diagnosis	n	Mean ± SD	Median (IQR)
Enchondroma	9	159.1 ± 40.1	168.6 (120.8–179.6)
ACT	13	296.6 ± 87.6	312.6 (214.6–375.6)
Grade 1 chondrosarcoma	14	380.9 ± 68.1	388.8 (321.4–437.3)
Grade 2 chondrosarcoma	19	546.6 ± 65.6	550.6 (498.5–580.6)
Grade 3 chondrosarcoma	12	761.2 ± 70.2	756.6 (737.0–805.4)
**Comparison**	Adjusted *p*-value *		
Enchondroma vs. ACT	1.000		
Enchondroma vs. Grade 1	0.112		
Enchondroma vs. Grade 2	**<0.001**		
Enchondroma vs. Grade 3	**<0.001**		
ACT vs. Grade 1	1.000		
ACT vs. Grade 2	**0.001**		
ACT vs. Grade 3	**<0.001**		
Grade 1 vs. Grade 2	0.059		
Grade 1 vs. Grade 3	**<0.001**		
Grade 2 vs. Grade 3	0.305		

Kruskal–Wallis test across all five groups: H = 59.30, *p* < 0.001. * Dunn’s pairwise post hoc test with Bonferroni correction for 10 comparisons; bolded values reached statistical significance (*p* < 0.05). Abbreviations: ACT, atypical cartilaginous tumor; IQR, interquartile range; PIV, pan-immune-inflammation value; SD, standard deviation.

## Data Availability

The data presented in this study are available on request from the corresponding author. The data are not publicly available due to privacy and ethical restrictions.
